# Multiple losses of sex within a single genus of Microsporidia

**DOI:** 10.1186/1471-2148-7-48

**Published:** 2007-03-29

**Authors:** Joseph E Ironside

**Affiliations:** 1 Institute of Biological Sciences, University of Wales, Aberystwyth, Ceredigion, UK

## Abstract

**Background:**

Most asexual eukaryotic lineages have arisen recently from sexual ancestors and contain few ecologically distinct species, providing evidence for long-term advantages of sex. Ancient asexual lineages provide rare exceptions to this rule and so can yield valuable information relating to the evolutionary forces underlying the maintenance of sex. Microsporidia are parasitic, unicellular fungi. They include many asexual species which have traditionally been grouped together into large, presumably ancient taxonomic groups. However, these putative ancient asexual lineages have been identified on the basis of morphology, life cycles and small subunit ribosomal RNA (*16S rRNA*) gene sequences, all of which hold questionable value in accurately inferring phylogenetic relationships among microsporidia.

**Results:**

The hypothesis of a single, ancient loss of sex within the *Nosema*/*Vairimorpha *group of microsporidia was tested using phylogenetic analyses based on alignments of *rRNA *and *RPB1 *gene sequences from sexual and asexual species. Neither set of gene trees supported ancient asexuality, instead indicating at least two, recent losses of sex.

**Conclusion:**

Sex has been lost on multiple, independent occasions within the *Nosema*/*Vairimorpha *group of microsporidia and there is no evidence for ancient asexual lineages. It appears therefore that sex confers important long-term advantages even upon highly simplified eukaryotes such as microsporidia. The rapid evolution of microsporidian life cycles indicated by this study also suggests that even closely related microsporidia cannot be assumed to have similar life cycles and the life cycle of each newly discovered species must therefore be completely described. These findings are relevant to the use of microsporidia as biological control agents, since several species under consideration as potential agents have life cycles that have been incompletely described.

## Background

Unravelling the interplay of forces that underlie the evolution of sex in eukaryotes remains one of the most elusive goals of evolutionary biology. Sexual reproduction entails considerable short-term costs in comparison with asexual reproduction, yet most eukaryotic organisms remain capable of sex. A large number of competing hypotheses have been proposed to explain this phenomenon (see [1-5] for reviews), all of which postulate long-term selective advantage of sex over asex. The hypothesis that sex has long-term benefits coupled with short-term costs has also been used to explain the observation that most asexual eukaryotic lineages are of recent origin and contain relatively few species [6]. This general pattern has been observed so frequently among animals and plants that ancient asexual lineages containing ecologically diverse species are regarded as highly exceptional [7].

Unlike plants and animals, most sexual fungi are isogamous and so do not suffer from the "two-fold cost" of producing male gametes that do not contribute resources to the offspring [8]. All sexual fungi are also able to reproduce asexually, allowing the direct cost of sexual reproduction to be minimised by engaging in sex only when conditions are optimal [9]. Furthermore, experiments have demonstrated that sexual strains of the yeast *Saccaromyces cerevisiae *are able to out-compete asexual strains under a range of environmental conditions [10,11]. Given the demonstrable benefits and apparently low costs of sex in fungi, one might reasonably predict that fungi should demonstrate a high incidence of sexuality. Indeed population genetics studies using molecular markers have revealed genetic signatures of sex in most fungal species that have been tested, several of which were previously thought to be asexual [12]. However, ancient asexual lineages also exist among the fungi. In the arbuscular mycorrhizal (AM) fungi, an ancient and diverse group of plant symbionts, ancient asexuality was indicated by the relaxation of concerted evolution acting upon multi-copy ribosomal RNA (*rRNA*) genes [13,14].

**Figure 1:**
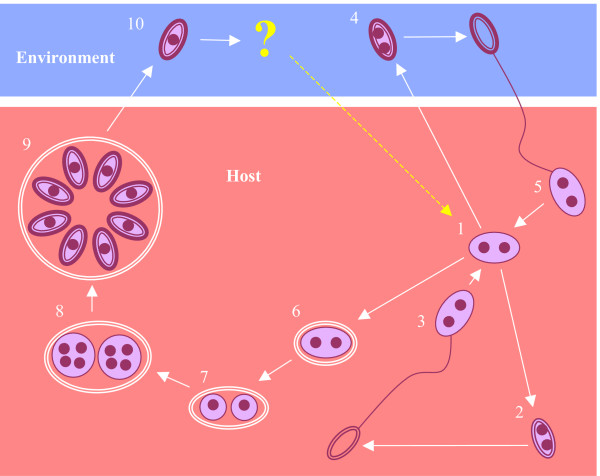
***Nosema/Vairimorpha *life cycles**. 1. Diplokaryotic meront 2. Primary (autoinfective) diplokaryotic spore 3. Within-host transmission or vertical transmission 4. Secondary (environmental) diplokaryotic spore (not *N. granulosis*, *N. empoascae*) 5. Horizontal transmission 6. Formation of sporophorous vesicle (*Vairimorpha *only) 7. Meiosis and karyokinesis 8. Nuclear division to form plasmodia 9. Monokaryotic sporogony 10. Monokaryotic environmental spore. Sexual species undergo stages 1–10, producing diplokaryotic mitospores and monokaryotic meiospores. The fate of meiospores has never been directly observed in *Nosema*/*Vairimorpha *but is assumed to be similar to that within the Amblyosporidae (Figure 2), in which monokaryotic spores differentiate into gametes and undergo cytoplasmic fusion, restoring the diplokaryotic state and completing the life cycle. In *V. imperfecta *monokaryotic sporogony is aborted at stage 9. Asexual lepidopteran *Nosema *species such as *N. bombycis *retain only the diplokaryotic cycles (stages 1–5). The life cycle of *N. granulosis *is further reduced, retaining only the primary diplokaryotic cycle (stages 1–3).

**Figure 2:**
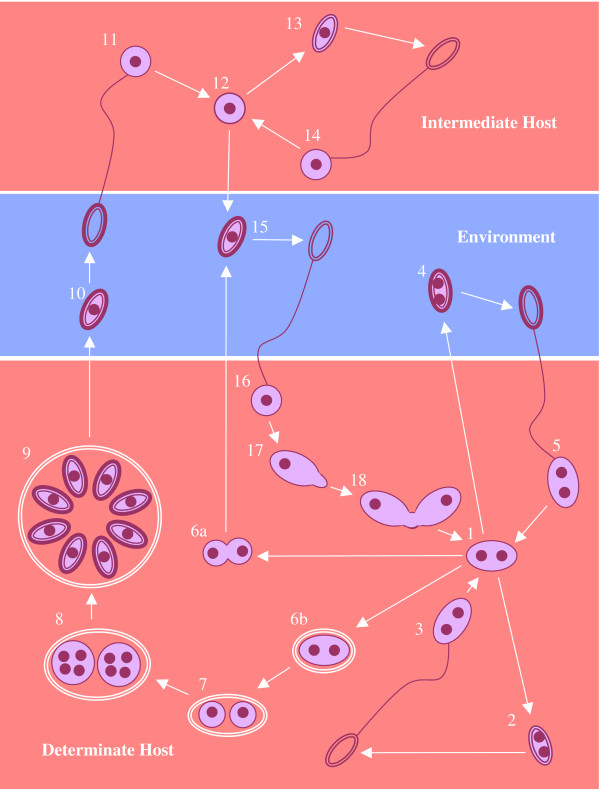
**Amblyosporidae life cycles**. 1. Diplokaryotic meront 2. Primary (autoinfective) diplokaryotic mitospore 3. Within-host transmission or vertical transmission (not *Hyalinocysta*) 4. Secondary (environmental) diplokaryotic spore (*Culicosporella *only) 5. Horizontal transmission 4a. Nuclear dissociation and karyokinesis (*Culicospora *and *Edhazardia *only) 4b. Formation of sporophorous vesicle (not *Culicospora*) 5. Meiosis and karyokinesis 6. Nuclear division to form plasmodia 7. Monokaryotic sporogony (abortive in *Culicosporella *and *Edhazardia*) 8. Monokaryotic meiospore 9. Transmission to intermediate host 10. Monokaryotic meront 11. Primary monokaryotic mitospore 12. Within-host transmission 13. Secondary monokaryotic mitospore (not *Culicosporella*) 14. Transmission to determinate host 15. Gametogenesis 16. Plasmogamy In the sexual *Amblyospora*, *Duboscqia*, *Hyalinocysta *and *Parathelohania *species, monokaryotic meiospores infect an intermediate host. Monokaryotic mitospores released by the intermediate host then infect the determinate host, where they differentiate into gametes and undergo plasmogamy to form diplokaryotic cells. In *Edhazardia aedis *and *Culicosporella lunata*, meiosis is abortive and any meiospores produced are non-functional while in *Culicospora magna*, meiosis is completely absent. *E. aedis *and *C. magna *produce monokaryotic mitospores by nuclear dissociation, eliminating meiosis. These monokaryotic spores are functionally equivalent to the spores produced by the intermediate host of *Amblyospora*, capable of infecting the determinate host and of undergoing gametogenesis and plasmogamy. In *C. lunata*, diplokaryotic mitospores are produced for horizontal transmission between larval hosts, eliminating both gametogenesis and plasmogamy.

The existence of ancient asexual lineages has also been implied in the microsporidia, a diverse group of intracellular fungal parasites that infect a wide range of vertebrate and invertebrate animal hosts. Some of the most intensively studied microsporidia belong to the genus *Nosema*, parasites of arthropods that have attracted attention both as causes of disease in honey bees and silk moths and as potential biological control agents for insect pests. Microsporidian life cycles typically involve the alternation of diplokaryotic (binucleate, diploid) stages and monokaryotic (uninucleate, haploid) stages (Figures 1 and 2). Members of the genus *Nosema *appear to have lost the monokaryotic cycle, remaining diploid throughout their life cycles, and are therefore considered to be asexual. Molecular evidence supporting asexuality in *Nosema *has been provided by variable rRNA gene sequences amplified from single spores of *N. bombi *[15], indicating relaxation of concerted evolution, a finding similar to that supporting asexuality in the AM fungi [13]. *Nosema *contains over 100 described species, with several new species added every year, and its host range spans the Arthropoda, including insects, arachnids and crustaceans. Furthermore, many of the physiological interactions between *Nosema *parasites and their hosts are complex and host-specific, indicating a high degree of host-parasite coevolution. These include many incidences of transovarial transmission [16] and, most dramatically, the feminization of genetically male crustacean hosts by the parasite *N. granulosis *[17]. Given the assumption of ancient asexuality within *Nosema*, the high level of species diversity and high degree of host-specialisation within this genus presents a major challenge to the doctrine of limited evolution within asexual eukaryotic lineages.

Morphologically based taxonomies of the microsporidia have generally assumed that changes of life cycle, such as the loss of the monokaryotic cycle, correspond to major evolutionary transitions [18-21]. It was on this basis that all microsporidian species that remained diplokaryotic throughout their life cycles and lacked a sporophorous vesicle were placed within *Nosema *[19]. Recently, the taxonomy of *Nosema *has been extensively revised on the basis of phylogenetic analyses using the sequence of the small subunit ribosomal RNA (*16S rRNA*) gene. These molecular phylogenies have shown "*Nosema*" species scattered widely throughout the Phylum Microspora [22], suggesting that the monokaryotic cycle has been lost on many separate occasions. On the basis of *16S rRNA *phylogenies, several microsporidian species have been transferred from *Nosema *to newly created genera (e.g. *Antonospora locustae*, *Paranosema grylli*, *Brachiola algerae*), while several species formerly assigned to the genus *Vairimorpha *(including *Vairimorpha*'s type species, *V. necatrix*) have been tentatively placed within *Nosema *[23]. Following the notation of Baker et al. [23], I will henceforth refer to the monophyletic group containing the *Nosema *type species *N. bombycis *and the *Vairimorpha *type species *V. necatrix *as *Nosema*/*Vairimorpha*. Most of the "*Vairimorpha*" species within this group possess complete sexual life cycles, with alternating monokaryotic and diplokaryotic stages. The exception is *V. imperfecta*, in which meiosis is followed by an abortive monokaryotic sporogony, indicating an intermediate phase in the loss of sex [24]. The presence of these sexual species within *Nosema*/*Vairimorpha *suggests that the sexual, monokaryotic cycle has been lost since the origin of the genus, perhaps on several separate occasions.

**Figure 3:**
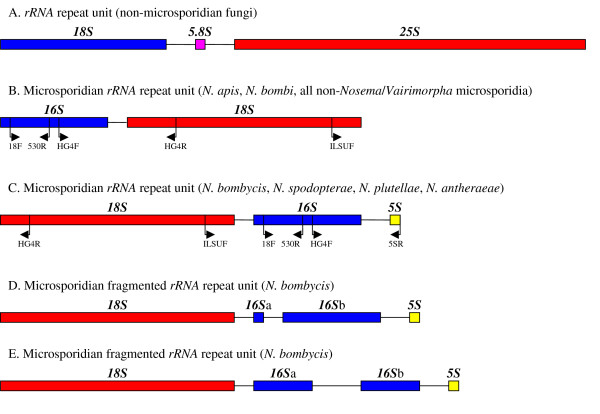
**Different types of *rRNA *repeat unit in microsporidia and in non-microsporidian fungi**. Figures 3B and 3C show the annealing sites of the primers used to determine the order of genes within microsporidian *rRNA *repeat units. Figures 3D and 3E show the fragmented *16S rRNA *genes discovered in *N. bombycis *by Iiyama et al. [41].

Phylogenetic analyses based solely upon *16S rRNA *sequences are, however, of limited use when comparing closely related microsporidian species. This is because the microsporidian small ribosomal RNA subunit is substantially shorter than those of other fungi and lacks many of the more variable regions [25-27]. In fact, both the small and large ribosomal RNA subunits of microsporidia are even shorter than those of most prokaryotes and consist of little more than a core of highly conserved sequences. Consequently, microsporidian *16S rRNA *sequence alignments rarely contain sufficient phylogenetically informative sites to unambiguously assign a topology to trees consisting of closely related species [24,28-30]. However, despite their short length and low sequence variability, the rRNA genes of *Nosema/Vairimorpha *vary exceptionally in the order in which they occur within the ribosomal repeat unit. In non-microsporidian fungi, and most other eukaryotes, the order of rRNA genes from 5' to 3' is *18S*, *5.8S*, *25S*, with internal transcribed spacers between the *18S *and *5.8S *subunit genes (*ITS1*) and between the *5.8S *and *25S *subunit genes (*ITS2*) (Figure 3a). In the *Nosema *species *N. apis *[31] and *N. bombi *[15] and in all known non-*Nosema *microsporidian species [32-36] the *18S *subunit is reduced in size to *16S*, the *5.8S *and *25S *subunit genes are reduced and the *ITS2 *spacer is absent, producing the gene order 5'-*16S*, *18S*-3' (Figure 3b). In *N. bombycis *an additional rearrangement has occurred, placing the *18S *subunit gene upstream of the *16S *subunit gene [37]. A *5S *subunit is also positioned downstream of the *16S *subunit of *N. bombycis *to give the order 5'-*18S*, *16S*, *5S*-3' (Figure 3c) [37]. This deviates from the arrangement seen in the model microsporidium *Encephalitozoon cuniculi*, in which no *5S *subunits occur in the vicinity of the larger ribosomal repeat unit [38]. The unusual ribosomal subunit gene order demonstrated by *N. bombycis *also occurs in *N. spodopterae *[39], *N. plutellae *(direct submission to Genbank: AY960987) and *N. antheraeae *[40], which, like *N. bombycis*, are parasites of Lepidoptera and which fall close to *N. bombycis *in phylogenetic trees based on the *16S rRNA *gene sequence [29]. *Nosema*/*Vairimorpha *species possess multiple copies of the ribosomal RNA repeat unit which can show intragenomic variation in sequence [15,31] and some isolates of *N. bombycis *also possess fragmented copies of *rRNA *genes (Figure 3d, e) [41] which are transcriptionally active and coexist with intact *rRNA *copies within the same genome. The fact that, within *Nosema*/*Vairimorpha*, the rRNA repeat unit exists in multiple copies with intragenomic variation in gene order, integrity and sequence is a potential source of confusion in phylogenies based entirely upon *16S rRNA *gene sequences.

In order to improve the resolution of the *Nosema rRNA *gene phylogeny, the *18S rRNA *gene was sequenced from a representative group of sexual and asexual species to produce an alignment including both *16S *and *18S rRNA *gene sequences. As an independent test of the phylogeny indicated by the *rRNA *genes, the largest subunit of the single-copy protein coding gene *RNA polymerase II *(*RPB1*) was also sequenced from each of the *Nosema*/*Vairimorpha *species. These two phylogenetic analyses supported incompatible hypotheses for speciation within *Nosema*, casting doubt upon the reliability of the *rRNA *gene sequence phylogeny. However, both phylogenetic hypotheses supported multiple, independent losses of sex within the *Nosema *genus. The implications of these results are discussed with regard to the evolution and maintenance of sex in microsporidia and to the evaluation of microsporidia as agents of biological control.

## Results

### rRNA Phylogeny

**Figure 4:**
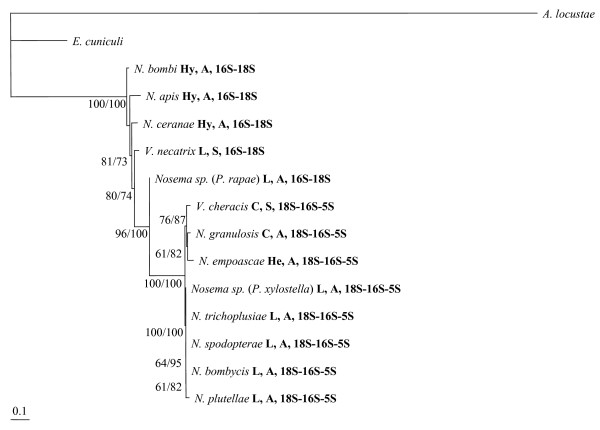
**Consensus phylogenetic tree for the *16S *and *18S rRNA *genes of *Nosema*/*Vairimorpha***. Maximum likelihood and Bayesian inference criteria produced identical consensus tree topologies. Support for each internal node is given as maximum likelihood bootstrap value/Bayesian posterior probability of partition. Branch lengths are drawn proportionally to the number of changes assigned to each branch. The notation following each species name indicates host (Hy = Hymenoptera, L = Lepidoptera, C = Crustacea, He = Hemiptera), life cycle (A = asexual, S = sexual) and *rRNA *gene order.

In order to improve the resolution of the *Nosema*/*Vairimorpha rRNA *phylogeny, both the *16S *and *18S *ribosomal genes were sequenced from a representative group of two sexual and five asexual *Nosema*/*Vairimorpha *species, including parasites of insect and crustacean hosts. To these were added six published sequences, obtained from Genbank (See Additional File 1). Phylogenetic analysis of the resulting DNA sequence alignment using maximum likelihood and Bayesian inference yielded identical consensus trees, both of which supported a phylogeny in which *Nosema*/*Vairimorpha *is divided into two well-supported clades (Figure 4). These two major clades correspond to the two different *rRNA *gene orders found within *Nosema*/*Vairimorpha*. Sexual species occur within both clades, indicating at least two independent losses of sex.

### RPB1 Phylogeny

**Figure 5:**
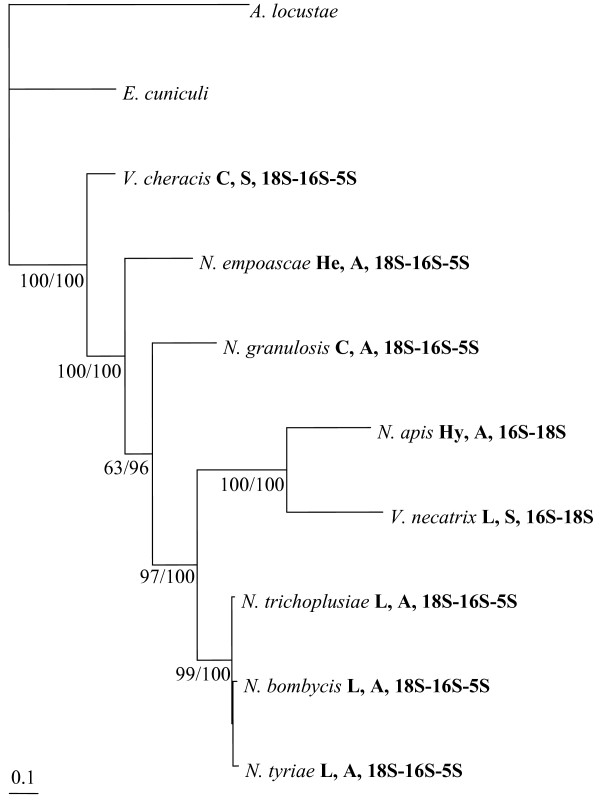
**Consensus phylogenetic tree for the *RPB1 *gene of *Nosema*/*Vairimorpha***. Maximum likelihood and Bayesian inference criteria produced identical consensus tree topologies. Support for each internal node is given as maximum likelihood bootstrap value/Bayesian posterior probability of partition. Branch lengths are drawn proportionally to the number of changes assigned to each branch. The notation following each species name indicates host (Hy = Hymenoptera, L = Lepidoptera, C = Crustacea, He = Hemiptera), life cycle (A = asexual, S = sexual) and *rRNA *gene order.

*RPB1 *occurs as a single copy in *V. necatrix *with a length of 1,606 codons (4818 bp), uninterrupted by introns [42]. A 1979 bp fragment of the *RPB1 *gene was sequenced from a representative group of two sexual and six asexual *Nosema*/*Vairimorpha *species, including parasites of insect and crustacean hosts. To these was added the published sequence of *N. tyriae*, obtained from Genbank (See Additional File 1). Phylogenetic analysis of the resulting DNA sequence alignment using maximum likelihood and Bayesian inference yielded identical consensus trees (figure 5). The topology of this *RPB1 *gene tree was incongruent with that of the consensus *rRNA *tree, indicating no correspondence between *rRNA *gene order and *RPB1 *gene sequence. However, despite its incongruence with the *rRNA *tree, the topology of the consensus *RPB1 *tree still indicated at least two independent losses of sex.

### Hypothesis Testing

**Figure 6:**
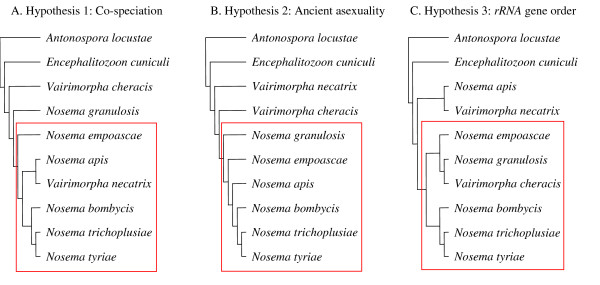
**Phylogenetic hypotheses for speciation within *Nosema*/*Vairimorpha***. Hypotheses were generated according to maximum criteria, using the following topological constraints: A. co-speciation: parasites of insects are constrained to be monophyletic, B. ancient asexuality: asexual species are constrained to be monophyletic, C. *rRNA *gene order: species with the 5'-*18S*, *16S*, *5S*-3' gene order are constrained to be monophyletic.

Approximately unbiased (AU) and weighted Shimodaira-Hasegawa (WSH) tests [43,44] were performed upon the *rRNA *and *RPB1 *alignments in order to compare the likelihoods of three different phylogenetic hypotheses for *Nosema*/*Vairimorpha *speciation. This was accomplished by comparing trees generated according to maximum likelihood criteria, under topological constraints corresponding to a priori hypotheses of 1) host-parasite co-speciation (insect parasites are monophyletic), 2) ancient asexuality (asexual species are monophyletic), 3) a single fixation of the 5'-*18S, 16S, 5S*-3' gene order (species with the 5'-*18S, 16S, 5S*-3' gene order are monophyletic) (Figure 6). Results of the tests are given in table 1.

**Table 1:** Results of approximately unbiased (AU) and weighted Shimodaira-Hasegawa (WSH) tests, comparing three phylogenetic hypotheses for *Nosema*/*Vairimorpha *speciation.

Gene	Constraint (monophyly)	-ln L	Diff in -ln L	AU test P	WSH test P
*RPB1*	Insect parasites	13940.79			
	*rRNA *gene order	14004.82	64.03	0.001	0.001
	Asexuality	14097.82	157.03	<0.0001	<0.0001
					
*rRNA*	*rRNA *gene order	16981.85			
	Insect parasites	17052.06	70.21	0.0002	<0.0001
	Asexuality	17969.55	987.70	<0.0001	<0.0001

The constrained *rRNA *tree produced by the hypothesis of a single change in *rRNA *gene order was completely congruent with the maximum likelihood *rRNA *consensus tree (Figure 4). The comparison of constrained *rRNA *trees indicated that the hypothesis of a single change in gene order was significantly more likely to reflect the true phylogeny than either the ancient asexuality hypothesis or the host-parasite co-speciation hypothesis (Table 1). In contrast, the comparison of constrained *RPB1 *trees indicated that the host-parasite co-speciation hypothesis was significantly more likely to reflect the true phylogeny than either the ancient asexuality hypothesis or the hypothesis of a single change in gene order. The host-parasite co-evolution tree and the maximum likelihood consensus tree were incongruent only with regard to the position of the crustacean parasite *N. granulosis*, which was weakly supported in the consensus tree. Both sets of gene trees indicated that the hypothesis of ancient asexuality was the least likely of the three alternative hypotheses (Table 1).

## Discussion

### Incongruence of rRNA and RPB1 Phylogenies

The consensus phylogenetic trees produced for the *rRNA *and *RPB1 *genes of *Nosema*/*Vairimorpha *are clearly incongruent. The *RPB1 *tree tentatively supports monophyly of the insect parasites and thereby supports the prediction that parasites should co-speciate with their hosts, especially in the case of parasites such as *Nosema*/*Vairimorpha *in which the relationship between host and parasite is very intimate [45]. In contrast, the *rRNA *tree indicates paraphyly for the insect parasites, with the crustacean and hemipteran parasites forming a sister clade to the lepidopteran group containing *N. bombycis*. These odd bedfellows share the unusual 5'-*18S*, *16S*, *5S*-3' gene order and so the *rRNA *tree appears to support a single origin for this gene order. However, this conclusion requires the dubious assumption that the sequences of *rRNA *genes are independent of the *rRNA *gene order.

**Figure 7:**
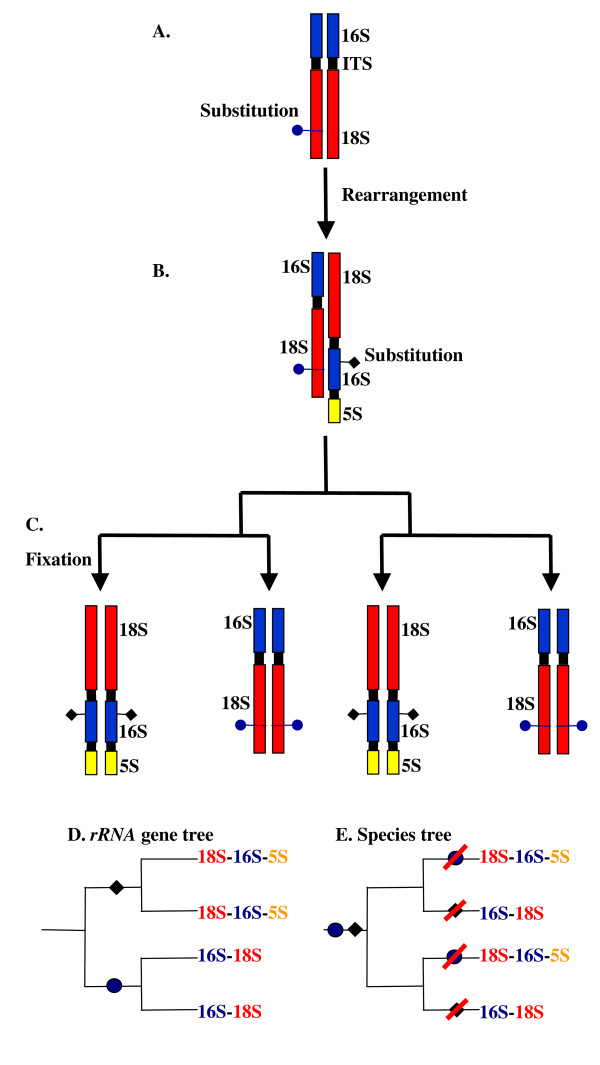
**Multiple, independent fixations of one *rRNA *copy can produce an *rRNA *phylogenetic tree that is incongruent with the species tree**. A-B: two copies of the *rRNA *repeat unit accumulate differences in nucleotide sequence and in gene order. C: speciation occurs and one *rRNA *repeat unit copy is fixed within each new species. D-E: each *rRNA *repeat unit copy is fixed twice, independently, resulting in an *rRNA *gene tree that is incongruent with the species tree.

The *Nosema*/*Vairimorpha rRNA *repeat unit occurs as multiple copies [15,31] and there is evidence for intragenomic variation in the sequence of the *rRNA *genes, indicating relatively low levels of concerted evolution [15,41]. It is therefore possible that *rRNA *repeat units with both gene orders coexisted in the *Nosema*/*Vairimorpha *genome for some time. If the genome of the common ancestor of *Nosema*/*Vairimorpha *contained some *rRNA *repeat units with the 5'-*18S*,*16S*,*5S*-3' gene order and others with the 5'-*16S*, *18S*-3' gene order, it would have been possible for the 5'-*18S*, *16S*, *5S*-3' gene order to have been lost or fixed on several independent occasions within the genomes of different *Nosema*/*Vairimorpha *species (Figure 7). Nucleotide substitutions that occurred after the rearrangement that produced the 5'-*18S*, *16S*, *5S*-3' gene order but before speciation from the common ancestor would, in this case, be shared across species by all 5'-*18S*, *16S*, *5S*-3' *rRNA *repeat units. Such shared nucleotide polymorphisms would then be fixed or lost along with the gene order with which they were associated. Under this scenario, *Nosema*/*Vairimorpha *phylogenies based upon the *rRNA *sequence would falsely support monophyly of all species sharing the 5'-*18S*, *16S*, *5S*-3' gene order. In contrast, *RPB1 *is a single-copy gene and so its phylogeny is not likely to have been confused by the presence of different copies with different evolutionary histories.

### Multiple Losses of Sex within *Nosema*/*Vairimorpha*

Despite the differences in topology between the *rRNA *and *RPB1 *phylogenies, Shimodaira-Hasegawa and Kishino-Hasegawa tests of both phylogenies conclusively rejected monophyly of the asexual *Nosema*/*Vairimorpha *species. The closest phylogenetic group to *Nosema*/*Vairimorpha *(according to *16S rRNA *trees) contains three species of microsporidian parasites of freshwater crayfish, erroneously placed within the genus *Thelohania *[46-48]. The life cycles of all three "*Thelohania*" species are similar to that of *V. necatrix *and *V. cheracis *(Figure 1), containing both monokaryotic and diplokaryotic phases, with evidence of meiosis. This suggests that the most recent common ancestor of *Nosema*/*Vairimorpha *and the "*Thelohania*" parasites of crayfish was sexual and that the monokaryotic cycle, and hence the capacity for sexual reproduction, was lost on at least two occasions within the *Nosema*/*Vairimorpha *group. It can therefore be concluded that *Nosema*/*Vairimorpha *is not an ancient asexual lineage but consists of a core of sexual species that have repeatedly given rise to asexual clones. This situation is not uncommon among the fungi [49]. Many asexual strains of otherwise sexual fungal species have been isolated from environmental and medical sources [50,51] and laboratory studies have demonstrated that the capability for sexual reproduction can be rapidly eroded when fungi are constrained to reproduce asexually [52]. When the loss of sex occurs due to the deletion or inactivation of genes necessary for meiosis or sexual interactions, reversion to sex is considered to be unlikely [7] since this would generally require multiple, specific back-mutations.

The phylogenetic group including the crayfish "*Thelohania*" and *Nosema*/*Vairimorpha *is one of only three microsporidian groups in which the production of sexual meiospores has been described in detail. The others are the dipteran parasites of the family Amblyosporidae [53] and the "*Thelohania*" parasites of *Solenopsis *ants [54]. According to *16S rRNA *trees, the sexual "*Thelohania*" parasites of ants are phylogenetically distant both from the true *Thelohania *and from the false "*Thelohania*" of crayfish but form a close sister group to the asexual microsporidia of the genus *Anncaliia *[55] indicating that members of the latter genus may also have recently abandoned sex. Within the Amblyosporidae, life cycle evolution also appears to have followed a very similar pattern to that within *Nosema*/*Vairimorpha*, with *16S rRNA *trees indicating independent losses of the sexual cycle in the species *Edhazardia aedis*, *Culicospora magna *and *Culicosporella lunata *[56]. Like *V. imperfecta*, *E. aedis *and *C. lunata *undergo abortive meiotic sporogony, suggesting relatively recent loss of the sexual cycle. Frequent losses of the sexual cycle therefore appear to be common to several microsporidian groups.

### Why give up sex?

For obligate parasites such as the microsporidia sex has the potential to increase the efficiency of directional selection upon the parasite and hence the speed with which the parasite can adapt to overcome the resistance of its host [49,57]. Furthermore microsporidia are isogamous and are therefore unaffected by the "two-fold cost" of sex. Given these apparent advantages of the sexual cycle it is, perhaps, surprising that its loss appears to have occurred so frequently among the microsporidia. However, sex can carry additional costs arising from the production of gametes and the process of fertilisation. *Nosema*/*Vairimorpha *may be particularly affected by such costs because many species rely on efficient vertical transmission from female hosts to their offspring. Any additional energy costs incurred by sexual reproduction are passed on to the host, reducing its survival and/or fecundity and hence reducing vertical transmission of the parasite [58].

The situation is further complicated by the fact that the monokaryotic stages of sexual microsporidia perform other functions in addition to sex. Selection to retain monokaryotic stages of the life cycle may not, therefore, correspond directly to selection for the maintenance of sex. For example, meiospores produced by the sexual species *V. necatrix *and *V. plodiae *are very resistant to degradation in the extracellular environment. Production of meiospores in these species increases markedly at low temperatures [59,60], suggesting that meiospores may function as an overwintering stage, allowing the parasite to survive while active hosts are unavailable. In contrast, asexual *Nosema *parasites of Lepidoptera do not produce resistant meiospores but overwinter within the eggs, pupae or adults of the host and are vertically transmitted to the next generation. In this case, loss of the monokaryotic cycle may have been due to selection against the production of resistant spores rather than selection against sex.

Where the monokaryotic phase of the life cycle involves an intermediate host, as occurs in the Amblyosporidae [61] (Figure 2b), the absence or poor availability of this host might also select against the production of monokaryotic stages and hence against sex. The fate of meiospores produced by *V. necatrix *and other sexual *Nosema/Vairimorpha *species has never been documented and it is not known whether transmission is direct or involves an intermediate host. Similarly, the fate of meiospores is known in neither the crustacean *Thelohania *species nor the ant "*Thelohania*" species, leading to speculation that these sexual microsporidian groups might also use intermediate hosts [54,62]. If sexuality tends to be intimately associated with a multi-host life cycle in microsporidia then the loss of sex might occur as a by-product of the elimination of an intermediate host from the life cycle.

### Implications for Biological Control and Pest Management

In view of their status as pests and their potential for use in biological control, it is important to understand the sexual cycles of *Nosema/Vairimorpha *species and of microsporidia in general. There are now several documented cases of microsporidia being accidentally introduced to new geographical areas along with their hosts [63] and further, deliberate, introductions are contemplated for the purpose of biological control. Several of the proposed introductions involve sexual microsporidian species such as *V. necatrix*, *V. lymantriae *and *T. solenopsis *[64]. These examples are particularly worrying because the fate of the meiospores of these species is unknown. Previous studies have investigated the host range of microsporidian isolates with regard only to their determinate hosts [65,66]. There are, however, close similarities in meiospore production between *Amblyospora*, *Vairimorpha *and *Thelohania*. The possibility that meiospores of the latter two genera might, like those of *Amblyospora*, infect non-target intermediate hosts needs, therefore, to be taken into consideration by proponents of their use in biological control. The potential for introduced microsporidian species to hybridise with native species and the effects that such gene flow would have upon the host range and pathogenicity of these organisms also requires consideration. This is particularly relevant with regard to the introduction of *V. necatrix *to Europe, where there exist many native *Nosema*/*Vairimorpha *species, including pests such as *N. apis *and parasites such as *N. bombi *that infect threatened insect species [67]. In order to conduct appropriate experiments to investigate these issues, a more detailed understanding of the sexual phase of microsporidian life cycles is urgently required, including knowledge of mating types and intermediate hosts.

## Conclusion

Phylogenetic analysis indicates that sex has been lost within the genus *Nosema*/*Vairimorpha *on at least two separate occasions. It is therefore concluded that that this genus does not constitute or include an ancient asexual lineage. Instead, the evidence presented here suggests that the many asexual *Nosema*/*Vairimorpha *species have evolved recently from sexual ancestors.

There is currently a trend towards describing new microsporidian species on the basis of incomplete life cycle data or even solely on the basis of their *16S rRNA *sequences. The evidence presented here indicates that the life cycles of *Nosema/Vairimorpha *species can evolve very rapidly relative to evolution at the molecular level. This means that a newly discovered species will not necessarily share life cycle characteristics with a model species, even if their DNA sequences are extremely similar. The evidence presented here also suggests that *rRNA *sequences are unreliable indicators of the phylogenetic relationships of closely related species and so the phylogenetic information yielded by comparison of *rRNA *sequences is of limited value. On this basis, it is proposed that taxonomic descriptions of microsporidian species should describe every stage of the life cycle and should include molecular phylogenetic information based on the sequences of several genes.

The finding that life cycle reduction through the loss of sex has occurred on multiple occasions both in *Nosema*/*Vairimorpha *and in the Amblyosporidae has important implications for the way in which life cycle evolution within the microsporidia, and within the fungi in general, is viewed. The majority of described *Nosema*/*Vairimorpha *species possess a reduced, asexual life cycle and so description of the genus on the basis of one or a few species might have indicated that the entire group was asexual. Many putatively asexual groups of microsporidia and other fungi are described on the basis of one or a few species. It is therefore possible that their life cycles are truncated versions of the life cycles of recent, sexual ancestors, sexual descendents of which have yet to be described.

## Methods

### Species used and nucleic acid extraction

Complete ribosomal repeat unit sequences, including both the *16S *and the *18S rRNA *genes, are published for nine *Nosema*/*Vairimorpha *species. All of these were parasites of Lepidoptera or Hymenoptera and all are asexual (See Additional File 1). The only *Nosema*/*Vairimorpha *species for which *RPB1 *sequences are published are *V. necatrix *and *N. tyriae*, both of which are parasites of Lepidoptera. In order to include species from a more representative range of hosts and to include multiple sexual species, the complete ribosomal repeat unit and a fragment of the *RPB1 *gene were sequenced from the crustacean parasites *N. granulosis *and *V. cheracis*, from the hemipteran parasite *N. empoascae*, from the lepidopteran parasites *N. bombycis*, *N. trichoplusiae *and *V. necatrix *and from the hymenopteran parasite *N. apis*. The genomic DNA of *N. granulosis*, *N. apis*, *N. empoascae*, *V. cheracis *and *V. necatrix *were extracted from infected tissue samples stored in ethanol. The genomic DNA of *N. bombycis *and *N. trichoplusiae *was extracted from purified spores stored in ethanol. Genomic DNA extraction was performed using Qiagen's DNeasy^® ^DNA purification kit, according to the manufacturer's instructions.

### Determination of rRNA gene order, amplification and sequencing of *rRNA *genes

The order of *rRNA *genes in the ribosomal repeat unit of each species was ascertained by PCR using gene order specific primer combinations. The primer set HG4F: HG4R [31] produced a 947 bp product only when *rRNA *genes occurred in the 5'-*16S*, *18S*-3' order (Figure 3B), while the primer sets ILSUF: 530R [37,68] and 18F: 5SR [37,69] produced products of 870 bp and 1624 bp only when the rRNA genes occurred in the 5'-*18S*, *16S*, *5S*-3' order (Figure 3C). The ribosomal repeat unit was amplified with Invitrogen's recombinant Taq DNA polymerase, in 5 fragments with an annealing temperature of 50°C and an extension time of one minute. Direct sequencing was performed using BigDye^® ^terminators on an ABI 3100 high throughput sequencer. PCR and sequencing were performed using the species-specific primers (see Additional File 2).

### Amplification and sequencing of *RPB1*

*RPB1 *occurs as a single copy in *V. necatrix *with a length of 1,606 codons (4818 bp), uninterrupted by introns [42]. A large (2958 bp) fragment of *RPB1 *was previously sequenced from *N. tyriae *[70], providing a second sequence from species within the *Nosema*/*Vairimorpha *group. Alignment of these two sequences allowed conserved regions of *RPB1 *to be identified, facilitating the design of degenerate PCR primers with which to amplify *RPB1 *from additional species. These initial sequences were then used to design specific *RPB1 *primers (See Additional File 1) for each of the *Nosema*/*Vairimorpha *species included in the analysis. The 1979 bp *RPB1 *fragment used in the analysis was amplified with Invitrogen's recombinant Taq DNA polymerase, using the species specific primer combinations: *N. apis*:NaRPB1_1F:NaRPB1_4R, *N. bombycis *and *N. trichoplusiae*: NbRPB1_1F:NbRPB1_5R, *N. empoascae*: NeRPB1_1F:NeRPB1_3R, *N. granulosis*:NgRPB1_1F:NgRPB1_4R, *V. cheracis*:VcRPB1_1F:VcRPB1_4R, *V. necatrix*:VnRPB1_1F:VnRPB1_5R. All primer combinations used an annealing temperature of 50°C and an extension time of two minutes. Direct sequencing was performed using BigDye^® ^terminators on an ABI 3100 high throughput sequencer, with the species specific primers (see Additional File 2).

### Phylogenetic analysis

Due to the high level of DNA sequence divergence between microsporidia and other fungi [71,72], outgroup species were selected from within Phylum Microspora. The two species chosen as outgroups were *Encephalitozoon cuniculi*, a parasite of mammals and *Antonospora locustae*, a parasite of orthopteran insects. According to the phylogenetic analysis of Vossbrinck and Debrunner-Vossbrinck [28], *Encephalitozoon *is a close sister group to *Nosema*/*Vairimorpha *within the Class Terresporidia while *Antonospora *is more distantly related, falling within Class Aquasporidia.

Ribosmal RNA gene sequences were aligned using Clustal W with an equal transition: transversion ratio, a gap opening penalty of 15 and a gap elongation penalty of 6.66. Since the internal spacers of ribosomal repeat units with the 5'-*18S*, *16S*, *5S*-3' *rRNA *gene order are not homologous to those of units with the 5'-*16S*, *18S*-3' order [37], the spacers were excluded from the alignment. *RPB1 *DNA sequences were aligned on the basis of an *RPB1 *amino acid sequence template, implemented in the programme DAMBE [73]. The *rRNA *and *RPB1 *alignments were tested for saturation using the methods of Steel *et al*. [74] and Xia *et al*. [75], implemented in DAMBE. In neither case was there significant evidence for saturation so all polymorphic sites were included in the phylogenetic analyses. In order to test the possibility that some *RPB1 *sequences were those of pseudogenes, Ka/Ks values were calculated between each new *RPB1 *sequence and the well-characterised *RPB1 *sequence of *V. necatrix*. All pairwise comparisons produced Ka/Ks values within the range 0.05–0.18 (Table 2), indicating strong purifying selection upon all sequences used in the alignment and hence providing a good indicator that all sequences were those of functional genes.

**Table 2:** Ka/Ks values for each new *Nosema*/*Vairimorpha RPB1 *sequence, compared to that of *V. necatrix*.

Species pair	Non-synonymous positions	Non-synonymous differences	Ka	Synonymous positions	Synonymous differences	Ks	Ka/Ks
*N. apis*:*V. necatrix*	1437.58	229.25	0.1793	383.42	211.75	0.9999	0.1793
*N. bombycis*:*V. necatrix*	1434.67	259.58	0.2071	386.33	247.42	1.4426	0.1436
*N. empoascae*:*V. necatrix*	1427.75	250.17	0.1996	393.25	291.83	3.4155	0.0584
*N. granulosis*:*V. necatrix*	1427.58	262.58	0.2110	393.42	285.42	2.5655	0.0822
*N. trichoplusiae*:*V. necatrix*	1434.00	260.83	0.2083	387.00	249.17	1.4664	0.1420
*V. cheracis*:*V. necatrix*	1421.33	278.25	0.2269	399.67	372.75	-	-
*V. cheracis*:*N. empoascae*	1409.42	143.17	0.1091	411.58	283.83	1.8895	0.0577

Values of Ka/Ks considerably lower than 1 indicate relatively low levels of sequence polymorphism at non-synonymous sites, an indicator of strong purifying selection and hence of gene activity. The sequence of *V. cheracis *is too divergent from that of *V. necatrix *for Ka/Ks cannot be calculated, so an additional comparison is performed between *V. cheracis *and *N. empoascae*.

Likelihood comparison of evolutionary models, based on the Akaike information criteria in ModelTest [76], indicated that the General Time Reversible model with gamma-distributed rate variation across sites and a proportion of invariable sites (GTR+G+I) was most suitable for phylogenetic analysis of both the *rRNA *and *RPB1 *alignments. Phylogenetic analysis was performed separately upon the *rRNA *and *RPB1 *alignments using maximum likelihood, implemented in PAUP* 4b10 [77] and Bayesian inference, implemented in MrBayes [78]. For each alignment, a maximum likelihood consensus tree was generated by conducting a heuristic search and bootstrapping with 100 replicates. The *rRNA *tree (figure 4) indicated a relatively long branch length for the *A. locustae *outgroup sequence. In order to check that this long branch was not distorting the tree, additional maximum likelihood and Bayesian searches were conducted, in which *A. locustae *was either replaced with the alternative outgroup *Heterosporis anguillarum *(AF387331) or omitted entirely, leaving the single outgroup *E. cuniculi*. The trees produced by these additional searches had identical topologies to those produced using the original *E. cuniculi *and *A. locustae *outgroup pair and were supported by qualitatively similar bootstrap values.

### Hypothesis testing

Three a priori evolutionary hypotheses (Figure 6) were compared by generating constrained phylogenetic trees in PAUP* and selecting the tree with the highest likelihood value using the approximately unbiased (AU) test of Shimodaira [43] and the more conservative weighted Shimodaira-Hasegawa (WSH) test [44], both implemented in Consel [79].

Hypothesis 1 (Co-speciation) proposes co-speciation of parasites and hosts and constrains all insect-parasitizing *Nosema*/*Vairimorpha *to form a monophyletic group.

Hypothesis 2 (Sex/Asex) proposes that sex has been lost once within *Nosema*/*Vairimorpha *and constrains all asexual *Nosema *to form a monophyletic group.

Hypothesis 3 (*rRNA *gene order) proposes that the *rRNA *gene order has changed once from 5'-*16S*, *18S*-3' to 5'-*18S*, *16S*, *5S*-3' and constrains all *Nosema*/*Vairimorpha *with the 5'-*18S*, *16S*, *5S*-3' *rRNA *gene order to form a monophyletic group.

## Supplementary Material

Additional file 1 Sequences included in *rRNA *and *RPB1 *alignments. Species, host, life cycle, source and Genbank accession number for each microsporidian *rRNA *and *RPB1 *sequence. Click here for file

Additional file 2 Primers used for PCR and sequencing of microsporidian *rRNA *and *RPB1 *genes. Names and sequences of oligonucleotide primers Click here for file
