# Supplementary material for: Multiple losses of sex within a single genus of Microsporidia
Source: BMC Evol Biol. 2007 Mar 29;7:48. doi: 10.1186/1471-2148-7-48 (PMC1853083; doi:10.1186/1471-2148-7-48)
Supplement: Additional file 1 — Sequences included in rRNA and RPB1 alignments. Species, host, life cycle, source and Genbank accession number for each microsporidian rRNA and RPB1 sequence. [file 1471-2148-7-48-S1.doc]

| Species | Host | Life cycle | Source | Genbank Accessions | |
| --- | --- | --- | --- | --- | --- |
|  |  |  |  | *rRNA* (*16S, 18S*) | *RPB1* |
| *Nosema apis* | Hymenoptera | Asexual | E. Mahoney, Queens University, Belfast, UK | U97150 | DQ996230 |
| *Nosema bombi* | Hymenoptera | Asexual | Genbank Accession | AY741107 | - |
| *Nosema bombycis* | Lepidoptera | Asexual | J. E. Smith, University of Leeds, UK | AY259631 | DQ996231 |
| *Nosema ceranae* | Hymenoptera | Asexual | Genbank Accession | DQ078785 | - |
| *Nosema empoascae* | Hemiptera | Asexual | L. Solter, Illinois Natural History Survey, USA | DQ996238, DQ996237 | DQ996232 |
| *Nosema granulosis* | Amphipoda | Asexual | J. E. Ironside, University of Wales, Aberystwyth, UK | AJ011833, DQ996239 | DQ996233 |
| *Nosema plutellae* | Lepidoptera | Asexual | Genbank Accession | AY960987 | - |
| *Nosema sp.* (*Pieris rapae*) | Lepidoptera | Asexual | Genbank Accession | AY383655 | - |
| *Nosema sp.* (*Plutella xylostella*) | Lepidoptera | Asexual | Genbank Accession | AY960986 | - |
| *Nosema spodopterae* | Lepidoptera | Asexual | Genbank Accession | AY747307 | - |
| *Nosema trichoplusiae* | Lepidoptera | Asexual | American Type Culture Collection, USA | U09282, DQ996243 | DQ996234 |
| *Nosema tyriae* | Lepidoptera | Asexual | Genbank Accession | - | AJ278948 |
| *Vairimorpha cheracis* | Decapoda | Sexual | E. Moodie, University of New England, Australia | AF327408, DQ996240 | DQ996235 |
| *Vairimorpha necatrix* | Lepidoptera | Sexual | R. Downs, Central Science Laboratories, York, UK | DQ996241, DQ996242 | DQ996236 |
| *Encephalitozoon cuniculi* | Mammalia | Asexual | Genbank Accession | AJ005581 | NM_001040904 |
| *Antonospora locustae* | Orthoptera | Asexual | Genbank Accession, Antonospora locustaeDB | Antonospora locustaeDB | AF061288 |
