# Supplementary material for: Multiple losses of sex within a single genus of Microsporidia
Source: BMC Evol Biol. 2007 Mar 29;7:48. doi: 10.1186/1471-2148-7-48 (PMC1853083; doi:10.1186/1471-2148-7-48)
Supplement: Additional file 2 — Primers used for PCR and sequencing of microsporidian rRNA and RPB1 genes. Names and sequences of oligonucleotide primers [file 1471-2148-7-48-S2.doc]

| Species | Gene | Primer | Sequence |
| --- | --- | --- | --- |
| All | *rRNA* | 18F  1537R | 5'-CAC CAG GTT GAT TCT GCC-3'  5'-TTA TGA TCC TGC TAA TGG TTC-3' |
| *N. apis*,  *V. necatrix* | *rRNA* | HG4F  HG4R  NaLSU1F  NaLSU1R  NaLSU2F  NaLSU2R  NaLSU3F  NaLSU3R  NaLSU4F  NaLSU4R | 5'-GCG GCT TAA TTT GAC TCA AC-3'  5'-CGC CGA ATT AAA CTG AGT TG-3'  5'-TCT GAC GTG CAA ATC GAT GA-3'  5'-TGA TGC GGT ATA GGT ACG GAT-3'  5'-AAA AAG AAG TAG TGA ATC CGT-3'  5'-GAA TCA AAA AGC AGA GTC GC-3'  5'-TGT ATT GGG TGT TTG AAA AG-3'  5'-TAG AGC CTC ACT ATC ATG CCT-3'  5'-AGA GAT CCA TTG CCG GAT AA-3'  5'-AGC CTT GAA TGT AAT TGG AA-3' |
| *N. bombycis*,  *N. empoascae*,  *N. granulosis,*  *N. trichoplusiae,*  *V. cheracis* | *rRNA* | ILSUF  530R  5SR  NbLSU1F  NbLSU1R  NbLSU2F  NbLSU2R  NbLSU3F  NbLSU3R  NbLSU4F  NbLSU4R | 5'-TGG GTT TAG ACC GTC GTG AG-3'  5'-CCG CGG C(T/G)G CTG GCA C-3'  5'-TAC AGC ACC CAA CGT TCC CAA G-3'  5'-GGA TGT CAT AAC GAT GAA GAA-3'  5'-TTG GTC TTT CGC CAC TAT-3'  5'-CCC TTT GAA CTT AAG CAT ATC-3'  5'-TGT AAC ACG TTT CTT AGC GG-3'  5'-TGG TTC ACA GCG AAA TGT CT-3'  5'-GAG CCA ATC TTT ATC CCA AAG-3'  5'-CGC ATC AGG TGT CTC TGT TTT-3'  5'-CGT AGT TTT GCA ACA TAA TTG-3' |
| *N. apis* | *Rpb1* | NaRPB1_1F  NaRPB1_1R  NaRPB1_2F  NaRPB1_2R  NaRPB1_3F  NaRPB1_3R | 5'-CG(A/G) AAG TGT GTG TTT TTA TTG-3'  5'-TAT TG(C/T) ATG AGG (C/T)GA TTG CT-3'  5'-TTC C(A/T)G AAG CAA TC(A/G) CCT CA-3'  5'-GCA CCG ACA GTT TTT TTG TC-3'  5'-GAT GAA GAG GAT TTA GAA AAT G-3'  5'-GTT TCT GCA GTT TTA ATA GCT GTA TC-3' |
| *N. bombycis*  *N. trichoplusiae* | *Rpb1* | NbRPB1_1F  NbRPB1_1R  NbRPB1_2F  NbRPB1_2R  NbRPB1_3F  NbRPB1_3R  NbRPB1_4F  NbRPB1_4R  NbRPB1_5F  NbRPB1_5R | 5'-GGG CAT ATC GAA CTC TCT AA-3'  5'-AGC CAC ATG AAA TTG TAA AAG-3'  5'-CCT TAA GAA ATA CGA AAT GGA-3'  5'-TCA ATT AAA CGA AAG TCT TCC-3'  5'-AAT CGA CGA CCT TCT CTT CAT-3'  5'-ACA TGA TTT CTC CTC CGC AT-3'  5'-ATT CGT CCA ATG AAA GTC GG-3'  5'-GCA AAT CCA AAT GGA ATT CTC-3'  5'-TCC GGG TCT AAA GGA TCT TT-3'  5'-GGG TCA TCT GAG TAG CAG GTT-3' |
| *N. empoascae* | *Rpb1* | NeRPB1_1F  NeRPB1_1R  NeRPB1_2F  NeRPB1_2R  NeRPB1_3F  NeRPB1_3R | 5'-CCA AGA TCA AAA AGA TTC TGG -3'  5'-TTG GAC GTT AAA AGG CGT GA-3'  5'-CGA TGC CAA CAT CTC CCT A-3'  5'-TTG CCC GTC CAA AGT TGT TT-3'  5'-CAT TGA ATC TCC TGC CAT CA-3'  5'-TTT AAC AAG CCT TCG CTG GA-3' |
| *N. granulosis* | *Rpb1* | NgRPB1_1F  NgRPB1_1R  NgRPB1_2F  NgRPB1_2R  NgRPB1_3F  NgRPB1_3R  NgRPB1_4F  NgRPB1_4R | 5'-ACT CTG AAT GCA CTC TGG GGA-3'  5'-TTC ATT TCA TCT CCA TCG AAA-3'  5'-AAA GAC ACA TGC AGG ATG GA-3'  5'-TAG CCT GTT GTT CTG GGC TTT-3'  5'-CGA CAA TGC TAA GAA TTC AGT AGA-3'  5'-CGA AGA CCT TAG AGA CTA AAT ATT C-3'  5'-AGC GTG TGT AGG ACA GCA GAA-3'  5'-CAC CAT CTC ATT GGG AGA ACA-3' |
| *V. cheracis* | *Rpb1* | VcRPB1_1F  VcRPB1_1R  VcRPB1_2F  VcRPB1_2R  VcRPB1_3F  VcRPB1_3R  VcRPB1_4F  VcRPB1_4R | 5'-TCC TGG AGT GCA TCT GCT TCT-3'  5'-ATT GAA GGG CGT GAT CTT CT-3'  5'-ACG CCA ACA TCT CCC TCG A-3'  5'-AAA TGT TCT CCC GAT CAT CC-3'  5'-AGG ATG ATC GGG AGA ACA TT-3'  5'-TGG TAG ATG TTC CCG TCA GC-3'  5'-TAC CAG TTC CAA TAC GGG GA-3'  5'-ACC CAA TTG AAG GCG CTT AT-3' |
| *V. necatrix* | *Rpb1* | VnRPB1_1F  VnRPB1_1R  VnRPB1_2F  VnRPB1_2R  VnRPB1_3F  VnRPB1_3R  VnRPB1_4F  VnRPB1_4R  VnRPB1_5F  VnRPB1_5R  VnRPB1_6F  VnRPB1_6R  VnRPB1_7F  VnRPB1_7R | 5'-CCT ACG TCG TAA ATA CAA TCA-3'  5'-TGC TCT CAA CAT TCC TTC CA-3'  5'-GTT CTT TTA GTT CCT CCT CC-3'  5'-TGT TGT AAC TTT GCG GCA TG-3'  5'-ATT TGT CTT GTG TCT CGC CCT-3'  5'-TCT CTC ATT GAC ATA CCT GG-3'  5'-TGT CAA TGA GAG AGA GCT TTG-3'  5'-TGG AAA TTA CAT GGA CTA GCA-3'  5'-GCT AGT CCA TGT AAT TTC CA-3'  5'-CCA CTC ATC TTT GTC TTT ATC-3'  5'-GTG GTA TTT ACA GAC AGA TGG-3'  5'-TCG GGC TCG TTG GAC TAT AA-3'  5'-TAT AGT CCC ACG AGC CCA ACA-3'  5'-TGC TTT CCT TTT CGT TTC CT-3' |
